# Facing Empathy: Reducing Facial-Differences Bias Through a Story

**DOI:** 10.1177/10556656261455305

**Published:** 2026-06-22

**Authors:** Brea Chouinard, Mary Cheuk, William Hodgetts, Jacqueline Cummine

**Affiliations:** 1 Computing Science, 3158 University of Alberta, Edmonton, Canada; 2 Psychology, 3158 University of Alberta, Edmonton, Canada; 3 Neuroscience and Mental Health Institute, University of Alberta, Edmonton, Canada; 4 Rehabilitation Medicine, 3158 University of Alberta, Edmonton, Canada

**Keywords:** counseling, peers, quality of life, social support

## Abstract

**Objective:**

This study investigated if an empathy-evoking vignette could reduce facial differences bias, while considering gender.

**Design:**

Participants (N = 124) were randomly assigned to read either an empathy-evoking vignette or nothing, after which they completed indirect and direct bias measures.

**Setting:**

The study was conducted entirely online.

**Participants:**

Those completing the study were members of the general public, none of which had a facial difference.

**Interventions:**

Participants either read a short, empathy-evoking vignette or nothing before then completing the tasks.

**Main Outcome Measures:**

The indirect measure of bias was an implicit association task. Explicit measures of bias were questions about preferences and attitudes toward people with facial differences.

**Results:**

Reading the vignette led to lower bias on indirect and direct measures in females, whereas in the small sample of males, vignette males tended toward greater bias than no-vignette males. Although we tried to expand insights into bias in different genders, we did not have enough non-binary participants.

**Conclusion:**

This study showed that empathy-evoking vignettes can attenuate facial differences bias in females but may inadvertently heighten bias in males, underscoring the moderating role of gender in narrative-based interventions. Future work should explore diverse narrative types and perspectives, using a wider range of stimuli (varied facial differences, genders, ethnicities) and participant genders, and should incorporate gender into analyses of bias.

People with facial differences are at high risk of experiencing negative stigmatization.^1–3^ Facial differences can be congenital, such as port-wine birthmarks, cleft-lip differences, moles, or haemangioma^4^; or acquired, via burns, car accidents, or treatments/procedures such as for head and neck cancer.^5,6^ In general, people (incorrectly) associate facial differences with negative personality traits, lower intelligence, and less social competence^7^; negative emotions toward people with facial differences include embarrassment, sympathy, disgust/repulsion, and hostility.^8,9^ People with facial differences face harassment and discrimination, which can affect mental health^7,10^ and other quality of life indicators such as employment.^2^ For adolescents, facial differences bias results in being stared at, verbally targeted, teased, engaged in fights, or made to feel unwelcome.^3,11^ Moreover, people with facial differences often internalize the stigma, leading to depression and difficulties with social interaction.^12^ Altogether, facial differences bias/stigma is primarily negative and harmful.

Bias and stigma are embedded within, and the result of, social structures. Daily interactions for marginalized populations are influenced by the beliefs that others hold about them and their abilities, encapsulated by “bias” and “stigma.” The terms are tightly interconnected, because average levels of individual bias are diagnostic of systemic issues/stigma in the given environment.^13,14^ Both “bias” and “stigma” indicate forces that consciously and/or subconsciously affect how we act with and toward others.

Studying disability as a social construct reminds us that societal values and structures, not inherent differences, dictate individual functioning.^15^ It follows, then, that shifting facial differences biases can improve lives. Some people already respond to facial differences with less negative emotions like neutrality^8^ or surprise.^9^ Furthermore, when a non-deficit-based lens is emphasized, people with facial differences report positive communication abilities, service to others, observational skills, inner strength, abilities to question society, and a valued social circle.^16^ Combining social-disability and non-deficit-based models can mitigate harm by fostering positive associations with difference, through increased exposure and experience. Our aim was to increase non-deficit-based/positive associations with facial differences using an empathy-evoking vignette.

Empathy definitions vary and can include multiple dimensions,^17,18^ including cognitive empathy, or theory of mind, which is *knowing* what someone else is feeling; and affective empathy, which is *feeling* what someone else is feeling.^19,20^ Some bias reduction methods induce empathy to help people understand and feel another's situation and emotions or perceive similarities between themselves and the target population.^21,22^ These methods include receiving education about the condition,^21,23^ direct contact with the discriminated population,^24–26^ or via personal stories of triumph or positive experiences.^27,28^ Additionally, reading fiction can improve theory-of-mind and other social-cognitive abilities^29–31^, especially perspective-taking stories (eg, vignettes) that describe personal thoughts and feelings.^23^ Altogether, evoking empathy can reduce bias and can be evoked via vignettes. We aimed to decrease facial differences bias using an empathy-evoking vignette that associated positive feelings and experiences with facial differences.

Bias can be measured directly and/or indirectly. Direct methods require that a person be conscious of their feelings, which they rate using self-report questionnaires or Likert scales.^32^ Problematically, this renders them susceptible to social desirability bias—that is, consciously or subconsciously supplying answers that will be viewed favorably by the researcher or make the respondent feel good about themselves.^33^ Further, even without conscious bias, subconscious beliefs reinforced by social pressures and structures can drive biased behavior,^34^ subconsciously and automatically.^2,35^ Thus, bias evaluation should include indirect measures.^36^ One such measure is an implicit association task, in which participants sort images and words.^37^ The task relies on the well-established finding that people sort related concepts faster than unrelated concepts, reflecting the strength of internalized associations between them.^38,39^ Implicit association tasks have been used to study racism,^21,40,41^ sexism,^42–44^ ageism,^45,46^ ableism,^47,48^ and other social constructs (for a review, see Greenwald et al^49^). Implicit association studies have captured facial differences bias^2,35,50^ and demonstrated a disconnect between indirectly measured bias and explicit facial differences perceptions.^50^ Thus, we used both direct and indirect measures to explore facial differences bias.

Research on gender differences in bias is complex, and results vary depending on bias type (eg, implicit or explicit) and context. Some evidence suggests that males report higher levels of conscious prejudice than females (see^51^ for examples) and hold more negative bias for different in-group and out-group patterns.^52,53^ However, there is also evidence that women show stronger in-group bias, but only in certain contexts (ie, toward other women). More importantly, we do not have sufficient information about bias in genders other than female and male due to limited inclusion of non-binary people^54^ and lack of inclusive language.^55^ Altogether, bias research needs improved gender-inclusivity to better explore the complexities of bias across the full spectrum of gender identities.

Here we make explicit our intentions to write an informative paper from a non-ableist perspective. First, we aim to avoid ableist language^56^; data gathering procedures used facial “disfigurement,” but this manuscript uses the more neutral facial “difference” (When necessary, “disfigurement” is in scare quotes to indicate the terminology used during data collection). Second, we expand gender consideration, using female, male, and non-binary.^55^ Third, we empower individuals with limited access to life chances by: including non-binary as a gender category, prioritizing “female” before “male” in dual-gender references, and emphasizing positive associations (preference, warmth, positive attributes) with facial differences. We intentionally presented our results with preference for, warmth toward, and positive attribution association with facial differences on the (traditionally) positive side of figures. These subtle, but meaningful changes shift the disability narrative from deficits to differences. To avoid reinforcing bias, we used strength-based language, recognizing that exposure to negative bias can exacerbate it both internally^57,58^ and externally^59,60^ to the identity group. As none of the authors have a facial difference, nor were people with facial differences consulted, all interpretations are from an allyship perspective.^61^ Future work will be improved by directly involving people with facial differences as part of, or consultants to, the research team.

This study aimed to determine the effectiveness of reading an empathy-evoking vignette that detailed experiencing, being accepting of, and having happiness and success in your life as a person with a facial difference. We hypothesized: (1) In those who had not read any vignette, females and gender minorities would exhibit less bias than males on both indirect and direct measures, consistent with established gender differences in bias^51,52^; (2) Vignette participants would have lower bias than no-vignette participants on indirect and direct measures, based on empathy's known bias-reducing effects^21,22^; and (3) Relationships between indirect and direct measures would be stronger in females and gender minorities than males, given research on gender differences in explicit measures and social desirability bias.

## Methods

### Participants

The study included participants over 18 years of age, recruited from the first author's university and the general public through listservs and social media postings. Consent was obtained online. After removing 1 outlier based on the indirect measure, 67 participants read an empathy-evoking vignette prior to the task (vignette group; age: mean = 25.0 years, range = 18-68 years; 53 female, 3 non-binary, 3 prefer-not-to-say) and 56 did not read anything (no-vignette group; age: mean = 29.9 years, range = 18-71 years; 40 female, 0 non-binary, 1 prefer-not-to-say). Participants were randomly assigned to vignette and no-vignette groups by the study software. There were more gender minorities (females, n = 93; non-binary, n = 3) than males (n = 24) overall (*X*^2^ = 44.041, *df* = 1, *P* < .01), but no differences in gender composition between the groups regardless of whether we considered all the categories or only “female” and “male” (Supplementary Info A, Table S1). There were no exclusion criteria.

### Materials

#### Empathy-Evoking Vignette

To shift the facial differences disability narrative, a vignette (Supplementary Info B) used second-person perspective (“You wake up…”) to immerse readers in the initial shock and emotional journey of acquiring a facial difference. The vignette focused on internal experiences, emphasized personhood beyond appearance, and explicitly stated that appearance doesn’t define worth (“Your face is forever changed, but that is no reflection of your personality”). By highlighting resilience, agency, success, and self-acceptance, the narrative aimed to dismantle negative stereotypes and promote a nuanced understanding of living with a facial difference.

In order to quantify the emotionality of the experimental vignette (ie, facial difference), we computed an emotional intensity score using the National Research Council Canada Emotional Intensity Lexicon (NRC-EIL^62,63^). The facial difference vignette was compared to a neutral vignette that was not used in this study, but was explicitly written for comparison (called “Walk In The Park”). Both vignettes were written to be easily understood by 13-15 year olds (Flesch-Kincaid grade level for Facial Differences = 8.58; for Walk In The Park = 8.59^64^). To compute emotional complexity scores, we extracted emotional intensity values for all words from each story that were represented in the NRC-EIL. Emotional intensity values range from 0 to 1, where 0 is low and 1 is high emotional intensity. The facial differences vignette had a greater percentage of emotional words (*P* = .01) and higher average emotional intensity of those words (*P* < .01) compared to the control story (See Supplementary Info B for information from both vignettes).

#### Implicit Association Task: Indirect Measure

Implicit association tasks require participants to sort image and word stimuli.^37^ Our images represented the trait categories “with” and “without facial differences” (see Supplementary Info C), and words represented “positive” and “negative” attribute categories. Each trial pairs different trait and attribute categories, yielding either stereotype-consistent (eg, face with facial difference; “misfit”) or stereotype-inconsistent (eg, face with facial difference; “happy”) trials. Training blocks familiarize participants with different assignments of stimuli and response sides (eg, associating “negative”/”positive” and “disfigured”/”non-disfigured” with the left or right response boxes), and test blocks gather response times to analyze. Bias is indicated by slower responses in stereotype-inconsistent compared to stereotype-consistent trials.

##### Stimuli

We used 10 photos (Supplementary Info C, Figure S1): 1 each of 5 different Caucasian men and then those same pictures each with a different digitally imposed facial difference (bruising, missing eye, enlarged nostril, port-wine birthmark, missing ear). We used 10 words: 5 positive (easy-going, happy, approachable, friendly, like) and 5 negative (misfit, lazy, grumpy, awkward, unfriendly). Word stimuli were matched for length, frequency, number of phonemes, and number of syllables (all *p*s > .29) to ensure response times reflected semantics, not basic word features.

##### D-Value

The implicit association task results in a D-value, which is the response time difference between stereotype-consistent and stereotype-inconsistent blocks, divided by the standard deviation of the respective blocks.^37^ D-values have magnitude and directionality: numbers further from zero indicate stronger / more bias, and polarity (ie, positive/negative) indicates bias for/against the stereotype. In our study, negative D-values represent bias against those with facial differences (more negative values represent stronger facial differences bias), and positive values represent bias against those without facial differences (higher values equating to more bias). In summary, facial differences bias is represented by negative numbers.

#### Explicit Bias Questions: Direct Measures

Explicit bias was assessed via 5 questions (Supplementary Info A, 6-10), adapted from Hartung et al,^50^ that were posed to participants after they had completed the implicit association task. Questions 6-8 used Likert scales, addressing preference for (people with/without “disfigurements”) and warmth toward (people with/without “disfigurements”). Question 9 measured association of positive attributes with “disfigurements” with methodological control; that is, using counterbalanced wording to ensure participants were reading and responding reliably. Question 10 presented 17 slider-based attribute pairs (eg, sad/happy) to assess association of positive attributes with “disfigurement”; negative attributes were always presented on the left, thus not controlling against potential social desirability bias.

##### Preference

Question 6 used a 7-point Likert scale (strongly preferring people with facial “disfigurements” to those without). Positive scores indicated preference for those with facial differences, near-zero no preference, and negative scores indicated preference for those without.

##### Warmth (Questions 7 and 8)

To assess relative warmth, we combined parallel items from warmth toward those with vs. without facial differences. Positive scores indicated more warmth toward those with facial differences, negative scores more warmth toward those without, and zero indicated equal warmth.

##### Controlled Attribute Association (Question 9)

Three inversely worded items were included to ensure careful responses. Association of positive attributes with facial differences yielded positive values, association of negative attributes yielded negative values, and near-zero indicated neutrality.

##### Uncontrolled Attribute Association (Question 10)

Consistent left-right placement of negative-positive attribute pairs facilitated social desirability bias, thus we refer to this question as “uncontrolled.” Association of positive attributes with facial differences yielded positive values, negative attributes yielded negative values, and near-zero indicated neutrality.

### Procedure

After reviewing and consenting to a 2-page form and standard consent questions, online participants (N = 124) were randomly assigned to read either an empathy-evoking vignette or nothing, after which they completed indirect and direct bias measures. Demographic information and other information known or hypothesized to influence bias were also collected and evaluated. Information such as education, income, country of residence, and size of their city were asked before completing the task. Information that may influence task performance, such as exposure to, or experience with, facial difference or disability were asked following the task. All responses were categorical (Supplementary Info A).

The indirect measure involved 7 blocks of picture and word sorting, preceded by familiarization and instructions. Afterward, participants completed explicit bias and demographic questions.

### Analysis

We first determined and removed any outliers based on the indirect measure in the vignette and no-vignette group separately. Demographic information was analyzed using chi-squared tests. Of note, all D-value averages by gender and vignette group were negative, that is, indicated facial differences bias. There were no groups or sub-groups scoring with bias against those without facial differences (ie, there were no positive averages). Thus, our analyses evaluated whether contrasts of interest had more or less facial differences bias.

#### Hypothesis 1. Baseline Bias Gender Differences

We evaluated baseline bias levels across genders using the no-vignette group only. There were no non-binary participants in this analysis. One-tailed, independent samples *t*-tests determined whether females scored with less bias (ie, D-values closer to zero), had higher preferences for, warmth toward, and positive associations with facial differences compared to males. If either group was not normally distributed, we used the appropriate non-parametric alternative for comparing 2 independent groups; the Mann-Whitney U test. The test used is noted when reporting results (eg, “*t”* for the parametric test and “*Mann-Whitney”* for the non-parametric test).

To further characterize the relationship between gender and bias, we carried out 1-tailed *t-*tests evaluating whether scores for each gender were significantly different from zero (where zero represents neutral). For each test, if the assumption of normality was not met, we used the Wilcoxon test as a non-parametric alternative. The test used is noted when reporting results (eg, “*t”* for the parametric test and “*Wilcoxon”* for the non-parametric test).

#### Hypothesis 2. Vignette Readers Will Exhibit Less Bias

##### Indirect Measure: D-Value

There were not enough non-binary participants to analyze as a separate group, so we first ran an independent samples *t-*test including all participants. Subsequently, we ran an ANOVA with gender (female; male) and group (vignette; no-vignette) as between-group factors, excluding data from 3 non-binary and 4 prefer-not-to-say participants. If there were any violations of normality, we used Greenhouse-Geisser corrections and noted that in the text.

##### Direct Measure: Explicit Bias Questions

For each of the 4 explicit measures (ie, preference for; warmth toward; controlled association of positive attributes; and uncontrolled association), we ran independent samples *t-*tests including all participants. Subsequently, we ran ANOVAs with gender (female; male) and group (vignette; no-vignette) as between-group factors. We also used 1-sample *t*-tests to determine whether ratings in each sub-group (female vignette, female no-vignette, male vignette, male no-vignette) were significantly different from zero/neutral. For the *t-*tests, if there were any violations of assumptions of normality, we substituted the Mann-Whitney U test in place of independent samples *t*-tests and the Wilcoxon test in place of the 1 sample *t-*tests. For each comparison, the statistic used is denoted in the text.

#### Hypothesis 3. Bias-Measure Relationships Across Genders

We performed correlations between D-values and the 4 explicit measures. This was carried out separately for females/gender minorities and males, who had or had not read the vignette, resulting in 4 correlations.

The effect sizes reported are those that correspond to the respective tests. For ANOVAs we report eta squared, where 0.01 is small, 0.06 is medium, and 0.14 is large. For *t-*tests we report Cohen's *d*, where 0.10 is considered small, 0.30-0.50 is considered medium, and >0.50 is considered large. For the *Wilcoxon* (matched rank biserial correlation) and *Mann-Whitney* (rank biserial correlation), 0.1 is considered small, 0.3 is medium, and 0.5 is large.

## Results

There was 1 outlier in the no-vignette group and no outliers in the vignette group. Data from the no-vignette participant were removed from all further analyses. There were no group differences on any of the demographic variables, except income: *P* = .05, uncorrected (Supplementary Info A, Table S1). To evaluate the effect of income, we ran a D-measure ANOVA with group (vignette, no-vignette) and SES category (<$50k, $50-100k, >$100k) as factors. There was no main effect of group (*P* = .10, η^2^ = 0.02) or SES category (*P* = .09, η^2^ = 0.04), and no group by SES category interaction (*P* = .40, η^2^ = 0.01).

### Hypothesis 1. Baseline Bias Gender Differences

When comparing across females (n = 40) and males (n = 15) in the no-vignette participants (Table 1), bias in males (−0.84) was numerically (but not significantly) inverse-to-hypothesis for the D-value, by being slightly less negative than bias in females (−0.86). However, all direct measures trended in the hypothesized direction, with higher uncontrolled association of positive attributes with those with facial differences in females compared to males (*P* = .04). When looking at whether direct measures of bias were significantly different from zero within each gender (Table 2), both females (*P* < .01) and males (*P* = .02) preferred people *without* facial differences. However, differences emerged in the remaining metrics. Females were neutral regarding warmth (*P* = .35), but males were warmer toward people without facial differences (*P* = .04). On controlled association of positive attributes for people with facial differences females were neutral (*P* = .28), but males trended toward associating negative attributes with people with facial differences (*P* = .06). Finally, females associated positive attributes with people with facial differences on the uncontrolled metric (*P* < .01), while males were neutral (*P* = .54). The evidence converges to indicate that in general, males have more facial differences bias than females.

**Table 1.** Evaluating D-Values Across No-Vignette Females (n = 40) and Males (n = 15) Only, One-Tailed Tests, to Determine Whether Males Scored with More Bias Than Females.

		Females, N = 40 mean (SD)	Males, N = 15 mean (SD)	Test Statistic	*P*-Value	Effect Size
Indirect	D-value	−0.86 (0.39)	−0.84 (0.38)	*t* = 0.157	.56	0.05
Direct	preference	−0.48 (0.75)	−0.67 (0.98)	*Mann-Whitney* = 322.000	.32	0.07
	warmth	−0.05 (1.3)	−0.60 (1.2)	*Mann-Whitney* = 350.500	.13	0.17
	positive attribute association (controlled)	−0.06 (0.66)	−0.28 (0.64)	*t* = 1.086	.14	0.33
	positive attribute association (uncontrolled)	0.65 (1.0)	0.11 (0.60)	*Mann-Whitney* = 392.500	.04*	0.31

For all metrics, closer to 0 represents less bias; negative values show preference for people *without* facial differences (direct measures) or negative bias against people with facial differences (indirect measure); and positive values show preference for people with facial differences. *P ≤ .05.

**Table 2.** In No-Vignette Participants Only-Within Genders; Determining Whether Female and Male Scores Were Significantly Different Than Zero/Neutral.

No-Vignette Group, Female and Male Participants Only (n = 55)							
	Gender	N	Mean	SD	Test Statistic	*P*-value	Effect Size
Preference	females	40	−0.48	0.75	*Wilcoxon* = 6.00	*P* < .01*	−0.91
	males	15	−0.67	0.98	*Wilcoxon* = 0.00	*P* = .02*	−1.00
Warmth	females	40	−0.05	1.3	*Wilcoxon* = 33.50	*P* = .35	−0.14
	males	15	−0.60	1.2	*Wilcoxon* = 2.00	*P* = .04*	−0.81
Positive attribute association (controlled)	females	40	−0.06	0.66	*t* = −0.583	*P* = .28	−0.09
	males	15	−0.28	0.64	*t* = −1.660	*P* = .06*	−0.43
Positive attribute association (uncontrolled)	females	40	0.65	1.0	*Wilcoxon* = 593.00	*P* < .01*	0.52
	males	15	0.11	0.60	*Wilcoxon* = 44.50	*P* = .54	−0.02

Note: for the Student *t*-test, effect size if given by Cohen's *d*. For the Wilcoxon test, effect size is given by the matched rank biserial correlation.

### Hypothesis 2. Comparing Vignette to No-Vignette Readers; Vignette Readers Will Exhibit Less Bias

#### Indirect Measure: D-Values

##### No Consideration of Gender

Here we consider all participants regardless of gender (n = 123) in order to compare scores across vignette and no-vignette participants. As illustrated in Figure 1A, there were less biased values in vignette (mean = −0.70, SD = 0.46) than no-vignette participants (mean = −0.85, SD = 0.38), *t*(121) = 1.99, *P* = .03, *d* = 0.36 (1-tailed) indicating that the empathy-evoking vignette led to significantly lower indirectly measured facial differences bias.

**Figure 1.**
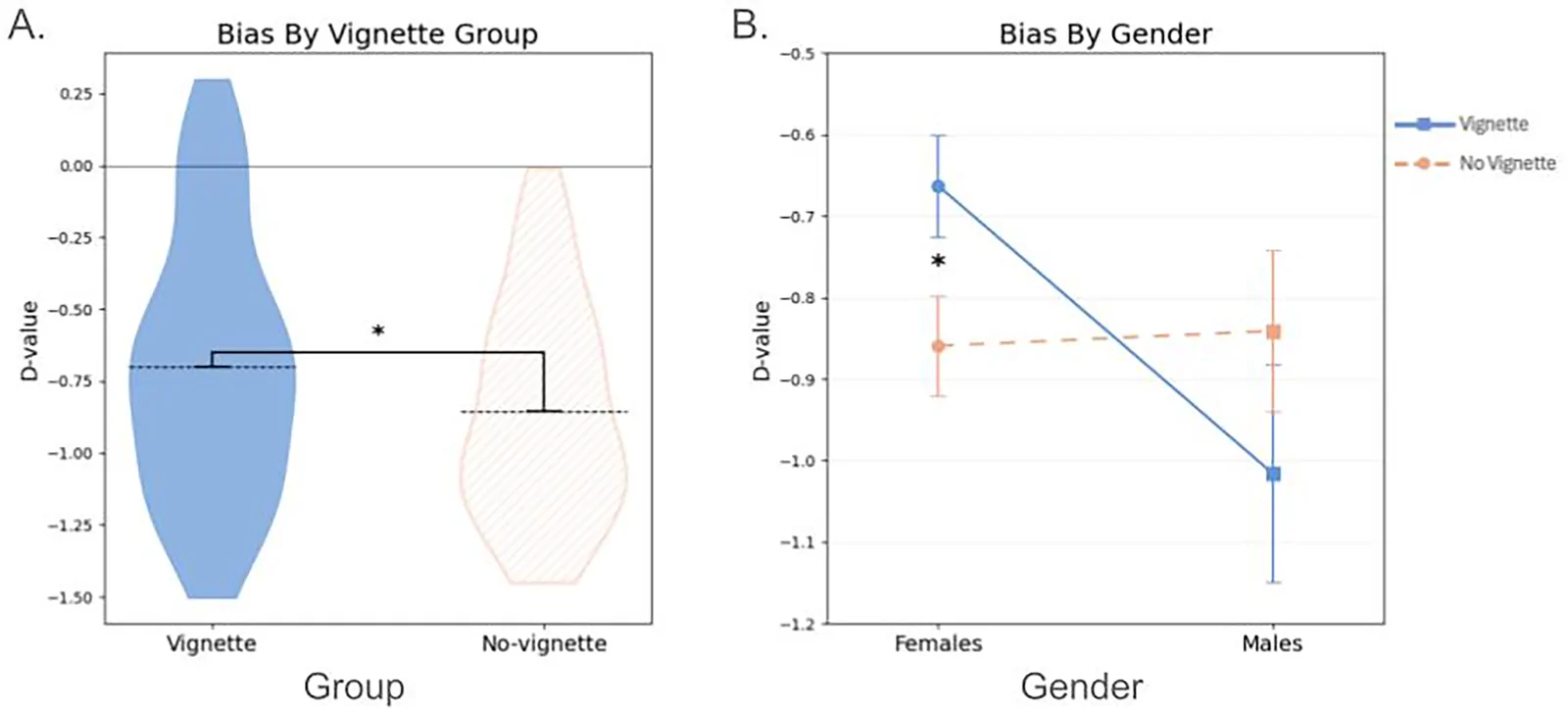
D-values across vignette and no-vignette groups without (1A) and with (1B) gender consideration. (1A) Violin plots indicating mean (hashed lines) and distribution for vignette (left violin) compared to no-vignette group (right violin). (1B) Female data points on left, male data points on right, solid line indicates vignette group. * = *P* < .05.

##### Considering Gender

Here we consider only females (n = 93) and males (n = 23), due to the low number of individuals in the other categories (n = 7). Note that an ANOVA that included income as an additional factor was not possible due to underrepresentation in 1 of the sub-groups (n = 1 in males, middle SES, vignette group). In the gender-by-group ANOVA (Figure 1B), there was no main effect of gender (*F*(1) = 2.722, *P* = .10, η^2^ = 0.02) or group (*F*(1) = 0.012, *P* = .91, η^2^ = 0.00), and only a borderline interaction (*F*(1,1) = 3.352, *P* = .07, η^2^ = 0.03). Further analysis revealed that vignette females (n = 53, mean = −0.66, SD = 0.45) scored with less bias (*t*(91)*,* 1-tailed = 2.205, *P* = .02, η^2^ = 0.46) than no-vignette females (n = 40, mean = −0.86, SD = 0.39). The same was not true for males (*Mann-Whitney,* 1-tailed = 41.00, *P* = .92, rank biserial correlation = −0.32); where vignette males (n = 8, mean = −1.02, SD = 0.38) showed more bias than no-vignette males (n = 15, mean = −0.84, SD = 0.38). Altogether, female vignette readers had less bias than no-vignette readers, but there was no difference in males.

### Direct Measures: Explicit Bias Questions

#### No Consideration of Gender

##### Preference

When considering all participants regardless of gender (n = 123), there was no difference (*Mann-Whitney,* 1-tailed = 1959.000, *P* = .32, rank biserial correlation = 0.00) between the vignette (n = 67, mean = −0.58, SD = 0.78) and no-vignette group (n = 56, mean = −0.54, SD = 0.81) for preference (Figure 2A). Thus, the groups were equally biased toward preferring people without facial differences. One-sample *t-*tests indicated that both groups scored significantly different from neutral (*Wilcoxon* = 11.5, *P* < .01, matched rank biserial correlation = −0.95 and *Wilcoxon* = 8.0, *P* < .01, matched rank biserial correlation = −0.94, respectively). Thus, participants in both the vignette and no-vignette groups, regardless of gender, expressed preference for people without facial differences.

**Figure 2.**
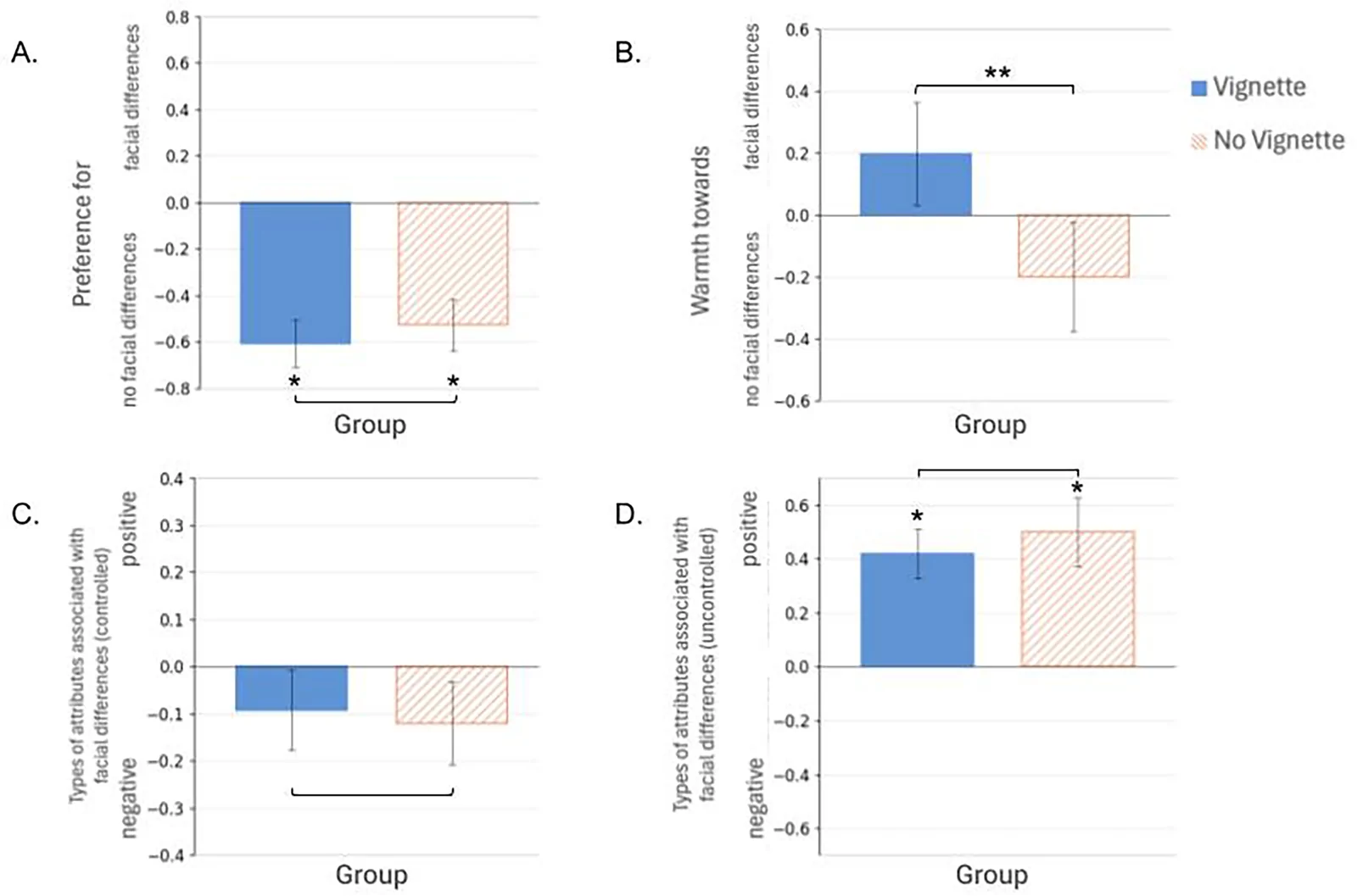
Directly measured bias between vignette (solid bars) and no-vignette (striped bars) groups. Positive values indicate preference for (Panel A), warmth for (Panel B), positive attribute association with (controlled (Panel C) and uncontrolled (Panel D)) people with facial differences. * = within-group difference from zero (*P* < .05). ** = between-group difference (*P* <.05).

##### Warmth

As illustrated in Figure 2B, there was a significant difference (*Mann-Whitney,* 1-tailed = 1572.00, *P* = .04, rank biserial correlation = −0.16) between the groups for warmth. Vignette participants reported warmer feelings toward people with facial differences than people without (mean = 0.18, SD = 1.25), while no-vignette participants reported warmer feelings toward people without facial differences (mean = −0.20, SD = 1.29). Although neither group's ratings were significantly different from zero (*P* = .08, matched rank biserial correlation = 0.32 and .09 matched rank biserial correlation = −0.36, respectively), vignette participants reported more warmth toward people with facial differences than no-vignette participants.

##### Controlled Attribute Association

As illustrated in Figure 2C, there was no difference (*Mann-Whitney* = 1747.5, *P* = .26, rank biserial correlation = −0.07) between vignette (mean = −0.08, SD = 0.64) and no-vignette participants (mean = −0.12, SD = 0.65) participants for the controlled measure of association of positive attributes with facial differences, and both groups’ scores were neutral (*P* = .29, matched rank biserial correlation = −0.09 and .09, matched rank biserial correlation = −0.18, respectively). When methodological control was in place, all participants associated neither positive nor negative attributes with facial differences.

##### Uncontrolled Attribute Association

As illustrated in Figure 2D, there was no difference (*Mann-Whitney* = 1809.0, *P* = .37, rank biserial correlation = −0.04) between vignette (n = 67, mean = 0.39, SD = 0.70) and no-vignette (n = 56, mean = 0.49, SD = 0.94) groups. Both groups significantly associated positive attributes with facial differences (*Wilcoxon* = 1368.0, *P* < .01, matched rank biserial correlation = 0.55 and *Wilcoxon* = 989.5, *P* < .01, matched rank biserial correlation = 0.38, respectively). Without methodological control, all participants significantly associated positive attributes with facial differences, suggesting potential social desirability bias.

### Considering Gender

#### Preference

The gender-by-group ANOVA included only participants who identified as female (n = 93) or male (n = 23), due to the low number of individuals in the other categories (n = 7). As illustrated in Figure 3A, there was a main effect of gender (*F*(1) = 8.047, *P* = .01, η^2^ = 0.07), a borderline effect of group (*F*(1) = 3.642, *P* = .06, η^2^ = 0.03), and a borderline interaction (*F*(1,1) = 3.335, *P* = .07, η^2^ = 0.03). One-sample *t-*tests indicated that both females (n = 93, *Wilcoxon* = 30.00, *P* < .01, matched rank biserial correlation = −0.92) and males (n = 23, *Wilcoxon* = 0, *P* < .01, matched rank biserial correlation = −1.00) showed a no-facial differences preference, but there was a stronger no-facial differences preference in males than females (*P* = .04, rank biserial correlation = 0.21). There was no difference in strength of preference (*Mann-Whitney* = 1788.5, *P* = .25, rank biserial correlation = 0) between vignette (mean = −0.61, SD = 0.80) and no-vignette (mean = −0.53, SD = 0.81) participants. The interaction indicated that while reading the vignette did not affect preference for females, vignette males (n = 8, mean = −1.38, SD = 1.1) had a borderline stronger no-facial differences preference (*Mann-Whitney* = 83.5, *P* = .06, rank biserial correlation = 0.39) than no-vignette males (n = 15, mean = −0.67, SD = 0.98). Altogether, both females and males preferred those without facial differences, but while reading the vignette had no influence on female preferences, it increased facial differences bias in males.

**Figure 3.**
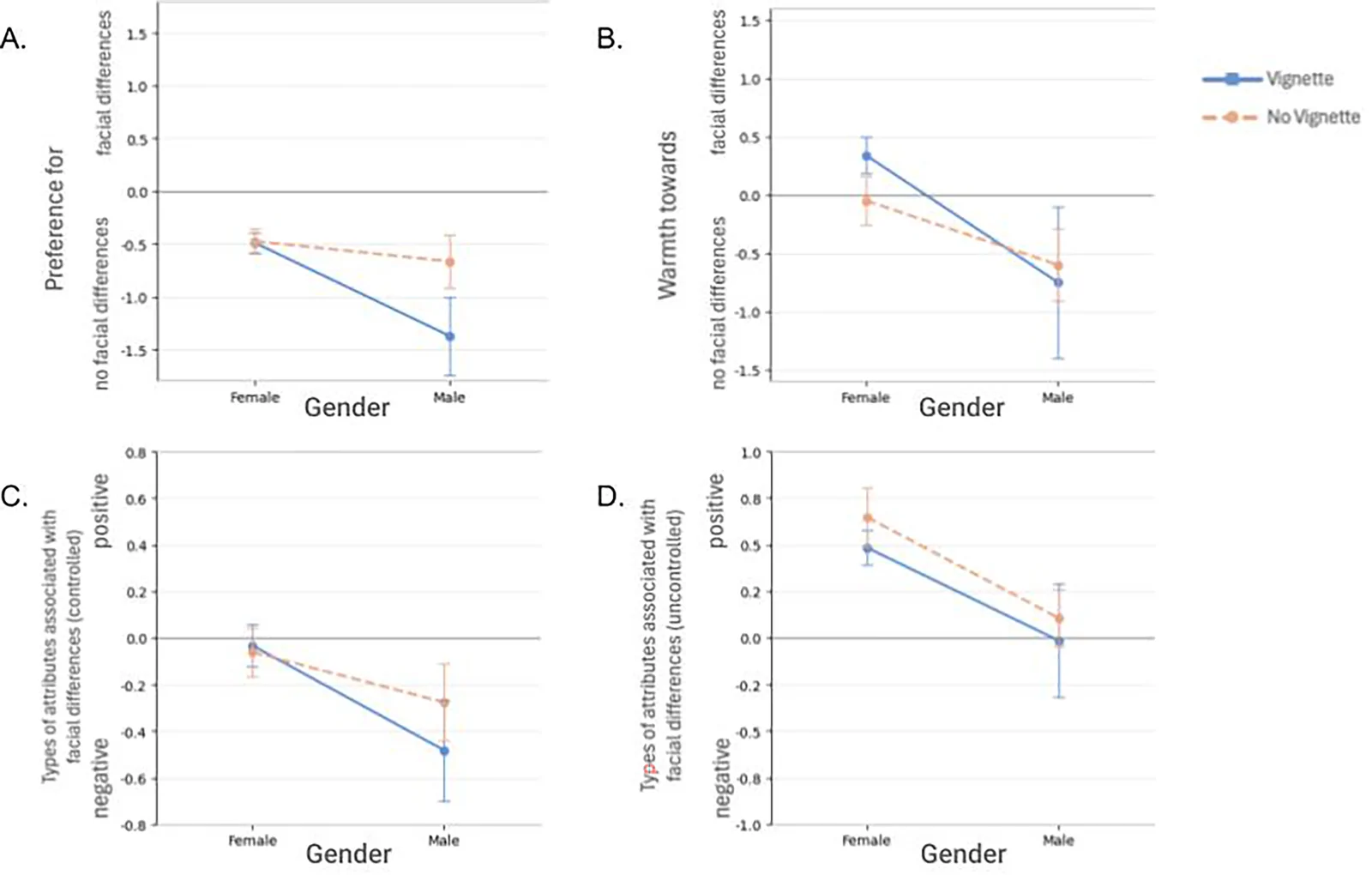
Directly measured bias in vignette (solid lines) and no-vignette (hashed lines) groups, with gender consideration. (3A) Vignette males more strongly preferred people without facial differences than no-vignette males. (3B) Vignette females reported more warmth toward people with facial differences than no-vignette females and males in both groups. (3C) Vignette males more strongly associated positive attributes with people without facial differences than all females. (D) Females more than males strongly associated positive personality traits with people with facial differences, regardless of group.

#### Warmth

The gender-by-group ANOVA included all participants who identified as female (93) or male (23). There was a main effect of gender (*F*(1) = 7.067, *P* < .01, η^2^ = 0.06), but no effect of group (*F*(1) = 0.151, *P* = .70, η^2^ = 0.00) or interaction (*F*(1,1) = 0.765, *P* = .38, η^2^ = 0.01). As illustrated in Figure 3B, on average, females reported warmer feelings toward those with facial differences (ie, positive scores), while males reported warmer feelings toward those without (ie, negative scores). Following up, vignette females (n = 53, mean = 0.34, SD = 1.16) reported significant warmth toward those with facial differences (*Wilcoxon* = 147.5, *P* = .02, matched rank biserial correlation = 0.55), where no-vignette females (n = 40, mean = −0.05, SD = 1.32) were neutral (*Wilcoxon* = 33.5, *P* = .68, matched rank biserial correlation = −0.14). In contrast, both groups of males reported large negative values, with no-vignette males (n = 15, mean = −0.60, SD = 1.18) scoring significantly warmer toward people without facial differences (*Wilcoxon* = 2.0, *P* = .04, matched rank biserial correlation = −0.81) (Note that vignette males (n = 8) scored with numerically more bias (−0.75) than the no-vignette males (−0.60), but due to the large standard deviation in the vignette group (SD = 1.83), the difference from zero was not statistically significant; *Wilcoxon* = 2.0, *P* = .17, matched rank biserial correlation = −0.60). Thus, in females, reading the vignette led to warmer reported feelings toward those with facial differences (decreased facial differences bias), while the difference in males was not universal enough to be measurable.

#### Controlled Attribute Association

The gender-by-group ANOVA contained all individuals who identified as female (n = 93) or male (n = 23). There was a main effect of gender (*F*(1) = 4.396, *P* = .04, η^2^ = 0.04), but no effect of group (*F*(1) = 0.313, *P* = .58, η^2^ = 0.00) or interaction (*F*(1,1) = 0.545, *P* = .46, η^2^ = 0.01). As illustrated in Figure 3C, females had less facial differences bias (ie, were closer to zero) compared to males regardless of whether they had read the vignette or not. Neither vignette (n = 53, mean = −0.03, SD = 0.66) *t*(52) = −0.358, *P* = .36, *d* = −0.05, nor no-vignette females (n = 40, mean = −0.06, SD = 0.66) *t*(39) = −0.583, *P* = .28, *d* = −0.09 significantly associated negative attributes with facial differences (*P* = 0.36, *P* = 0.28, respectively). However both vignette (n = 8, mean = −0.48, SD = 0.61) *t*(7) = −2.233, *P* = .03, *d* = −0.79 and no-vignette males (n = 15, mean = −0.28, SD = 0.64) *t*(14) = −1.660, *P* = .06, *d* = −0.43 associated negative attributes with facial differences. There were no differences between vignette and no-vignette readers on this measure, only between females and males.

#### Uncontrolled Attribute Association

The gender-by-group ANOVA contained only individuals who identified as either female (n = 93) or male (n = 23). There was a main effect of gender (*F*(1) = 6.929, *P* = .01, η^2^ = 0.06), but no effect of group (*F*(1) = 0.516, *P* = .47, η^2^ = 0.00) or interaction (*F*(1,1) = 0.012, *P* = .91, η^2^ = 0.00). As illustrated in Figure 3D, whether or not they read the vignette, females reported stronger associations between positive attributes and people with facial differences than males did (1-tailed, *Mann-Whitney* = 1497.00, *P* < .01, rank biserial correlation = 0.40). Both vignette ((n = 53, mean = 0.48, SD = 0.69) *Wilcoxon* = 987.0, *P* < .01, matched rank biserial correlation = 0.68) and no-vignette females ((n = 40, mean = 0.65, SD = 1.00) *Wilcoxon* = 593.0, *P* < .01, matched rank biserial correlation = 0.52) significantly associated positive attributes with facial differences, whereas vignette (n = 8, mean = −0.02, SD = 0.86) *t*(7) = −0.048, *P* = .52, *d* = −0.02 and no-vignette males (n = 15, mean = 0.11, SD = 0.60) *Wilcoxon* = 44.50, *P* = .54, matched rank biserial correlation = −0.02 were neutral (*P* = .52; *P* = .61). Again, there were no differences between vignette and no-vignette readers on this measure, only between females and males.

### Hypothesis 3. Bias-Measure Relationships Across Genders

Figure 4 illustrates relationships between the indirect and direct bias measures using a heat map. Neutral findings (ie, near zero) are gray, while positive relationships are indicated by progressively warmer colors (ie, oranges and reds) and negative relationships are indicated by progressively cooler colors (ie, light to dark blue). There were no indirect-direct correlations in vignette (all *p*s > .19) or no-vignette (all *p*s > .62) females, with 7 out of 8 *r*-values less than 0.10. However, among the direct measures, in vignette females, controlled association correlated with preference (*r* = 0.29, *P* = .03) and with uncontrolled association (*r* = 0.65, *P* < .01), and in no-vignette females, preference correlated with warmth (*r* = 0.36, *P* = .02), controlled association (*r* = 0.54, *P* < .01), and uncontrolled association (*r* = 0.42, *P* = .01). Thus, in females, regardless of reading the vignette or not, direct measures validly captured similar sentiments that were different to what was captured by the indirect measure.

**Figure 4.**
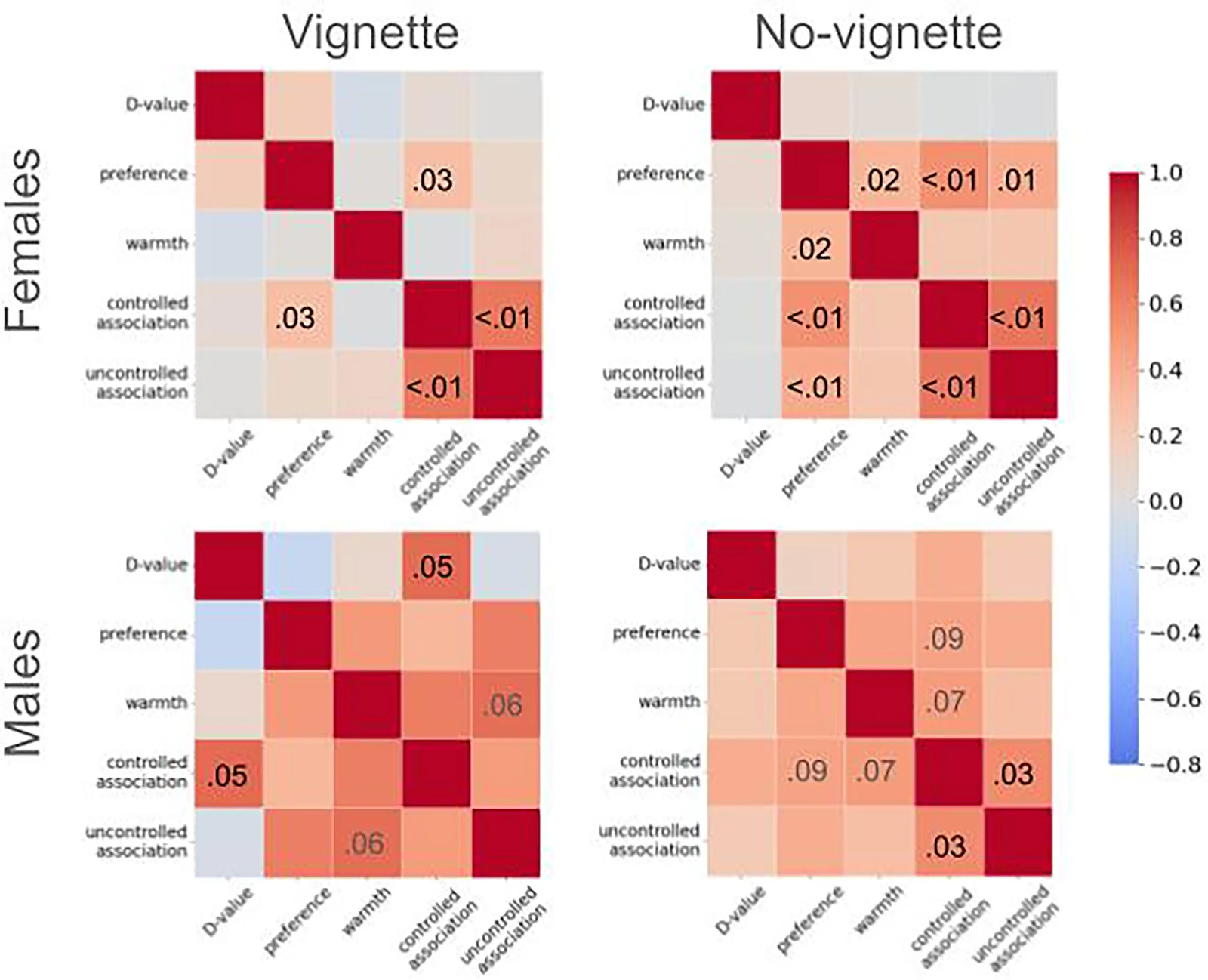
Heat maps for the 4 sub-groups of interest, from top right: vignette females (n = 53), no-vignette females (n = 40), vignette males (n = 8), and no-vignette males (n = 15). Font color represents significant (*P* < .05, black font) or near significant (.10 < *P* > .05, gray font) *P*-values within given cells.

In contrast, in vignette males (n = 8), there was an association between indirect and direct measures. D-values and controlled association were correlated (*r* = 0.70, *P* = .05), showing the relationship between stronger facial differences bias (indirectly measured) and associating negative attributes with facial differences (directly measured). Six out of 8 *r*-values were greater than 0.14. There was also a borderline correlation between warmth and uncontrolled association (*r* = 0.69, *P* = .06), suggesting a relationship between stronger facial differences bias and feelings of warmth toward those without facial differences. In no-vignette males (n = 15), the only correlation was between the controlled and uncontrolled association (*r* = 0.57, *P* = .03). Note that the male participant correlations are unreliable because we had so few male participants. Consequently, the limited data leave us uncertain about whether the indirect and direct measures were capturing the same information in males.

## Discussion

This study investigated if an empathy-evoking vignette could reduce facial differences bias, using indirect and direct measures, while considering gender. Although we did not have enough non-binary participants for statistical analyses, acknowledging diverse gender categories demonstrated a commitment to minimizing ableist and stereotype-reinforcing practices. Main findings indicated males generally exhibited more bias than females. Notably, the vignette resulted in lower indirectly measured bias and increased warmth toward those with facial differences in females only and appeared to increase bias in males. Implications of these findings are discussed below.

### Hypothesis 1. Baseline Bias Gender Differences

Our findings indicated greater bias against facial differences in males compared to females. Among no-vignette participants, males favored those *without* facial differences in preference and warmth, and associated negative attributes with people with facial differences. While these findings require cautious interpretation due to small sample size, they align with prior research indicating broader gender differences in prejudice.^51,52^ This pattern suggests that gender influences bias, highlighting the need for future studies to incorporate gender identity in bias and stigma research.

### Hypothesis 2. Vignette Readers Will Exhibit Less Bias

When gender was excluded from analysis, the results of indirect and direct measures were promising, though equivocal. There was less facial differences bias (*P* = .04) and significantly more warmth toward people with facial differences when comparing vignette and no-vignette participants. Although these suggestions of change resonate with foundational evidence that empathy can decrease bias toward stigmatized groups,^21,22,65–73^ they are not fully realized until we incorporate gender, limited despite our efforts to females and males. Although we tried to expand insights into bias in different genders, we did not have enough non-binary participants.

For females, reading the vignette led to lower bias on indirect and direct measures in females. This was supported by significant main effects of gender or interactions in the gender-by-group ANOVAs across all measures and follow-up tests indicating lower D-values and increased warmth toward people with facial differences when comparing vignette to no-vignette females. Additionally, neither vignette nor no-vignette females associated negative attributes with facial differences, unlike males. The vignette's effectiveness in reducing indirectly measured bias and increasing warmth in female participants aligns with studies on empathy induction^21,22^ and perspective taking,^23^ highlighting the potential of empathy-evoking narratives for implicit bias reduction, particularly in females.

Notably, our work found that employing empathy to decrease bias appeared to have the opposite-to-intended effect in males. In our small sample of males, vignette males tended toward greater bias than no-vignette males. Compared to no-vignette males, vignette males had a greater preference for people without facial differences than with (*Mann-Whitney* = 37.50, *P* = .04), associated negative attributes with facial differences (numerical only, not significant; *t* = 0.766, *P* = .23), and trended toward higher D-values (1-tailed; *Mann-Whitney* = 41.00, *P* = .09). This unexpected finding aligns with other research where interventions led to increased bias (eg, perspective-talking of a Black avatar leading to greater implicit race bias^74^). The inverse results may stem from the vignette's second-person language (“You wake up in the hospital…”). Since there is evidence that imagining how another person would feel evokes empathy, but imagining how you would feel evokes both empathy and distress,^75^ it is possible that our vignette evoked distress in males, unintentionally increasing bias. Alternatively, the vignette might have inadvertently reinforced in-/out-group identities in males, which is known to increase bias^76^ and decrease empathic brain response.^77^ Both the distress and out-group hypotheses might stem from the homogeneous (all white male) image stimuli.

Future research should explore how different narratives and perspectives impact bias across various demographic groups, particularly by diversifying visible identities (eg, skin color, sex, gender, ethnicity) in stimuli. Our work also emphasized the importance of considering gender as a moderator in bias reduction interventions. These caveats must be fully understood before widespread implementation of such empathy-based interventions.

### Hypothesis 3. Bias-Measure Relationships Across Genders

For females, there were positive relationships between direct measures in both vignette and no-vignette groups, but not between indirect and direct measures. This pattern of findings suggests that indirect and direct measures access different aspects of bias or potentially different constructs. While male data was insufficient to fully evaluate this hypothesis, the female findings suggest that multifaceted approaches may be necessary for measuring changes in bias.

### Limitations

There is some concern that participants can “cheat” on the implicit association task^78–81^. However, these studies involved explicit “cheating” instructions or practice, and converging evidence from our study does not support that participants were cheating. For example, vignette males tended toward more biased D-values than no-vignette males, and in vignette females, lower D-values occurred alongside greater warmth toward those with facial differences. Together, the evidence supports the validity of the D-value findings and thus the vignette's effectiveness in reducing bias.

Without a control vignette, we cannot definitively determine if the empathy-evoking vignette's specific content, or merely the act of reading, influenced results. Future research should use alternative vignettes to disentangle these factors, alongside exploring aspects like perspective and empathy dimension. Although we sought to expand understanding of biases across the gender spectrum, our sample's insufficient non-binary representation (n = 3) and disproportionate female-to-male ratio (n = 94 vs. n = 24) limited analyses. It's crucial to acknowledge the small male sample size, as the apparent lack of a modifying effect in males might reflect low power rather than a true absence of vignette impact. Future bias research must include and account for gender diversity.

We did not carry out a long-term follow-up, so we have no way to determine whether the effect was long-lasting. The online nature of the study could introduce uncontrolled variables. Future work should include those with facial differences on, or as consultants to, the study team.

### Future Directions

While not the focus of the current work, future studies that explore the impact of income and/or education level on facial differences bias as a function of gender and responsiveness to empathy invoking vignettes is needed, as the literature indicates that complex interactions among these variables may exist.^82^ For example, Mattan and Clousier^82^ explored pro-wealthy implicit bias and reported that men (but not women) showed increasing pro-wealthy implicit bias as a function of increasing income levels. In addition, their findings pointed to greater gender differences in pro-wealthy bias at lower education levels (regardless of income). The extent to which such interactions among these factors exist for facial differences bias is not yet known.

Our work strongly suggests future investigation into the differential effects of empathy-evoking narratives on bias, particularly across gender identities, using larger, balanced samples. Given the observed bias increase in male vignette readers, future research should explore diverse narrative types and perspectives, using a wider range of stimuli (varied facial differences, genders, and ethnicities) and participant genders. Having established the potential of empathy-evoking vignettes for reducing facial differences bias, long-term intervention effects on attitudes and behaviors can be examined. Crucially, to minimize harm and ableism, future research should actively involve individuals with facial differences in all research phases, from design through dissemination.

### Conclusion

This study showed that empathy-evoking vignettes can attenuate facial differences bias in females but may inadvertently heighten bias in males, underscoring the moderating role of gender in narrative-based interventions.

## Supplemental Material

sj-pdf-1-cpc-10.1177_10556656261455305 - Supplemental material for Facing Empathy: Reducing Facial-Differences Bias Through a Story Supplemental material, sj-pdf-1-cpc-10.1177_10556656261455305 for Facing Empathy: Reducing Facial-Differences Bias Through a Story by Brea Chouinard, Mary Cheuk, William Hodgetts and Jacqueline Cummine in The Cleft Palate Craniofacial Journal

## Data Availability

The participants of this study did not give written consent for their data to be shared publicly, so supporting data is not available.
