# Supplementary material for: Facing Empathy: Reducing Facial-Differences Bias Through a Story
Source: Cleft Palate Craniofac J. 2026 Jun 22;63(12):4199–212. doi: 10.1177/10556656261455305 (PMC13724823; doi:10.1177/10556656261455305)
Supplement: Supplementary file 1 [file sj-pdf-1-cpc-10.1177_10556656261455305.pdf]

Supplementary Figure S1.

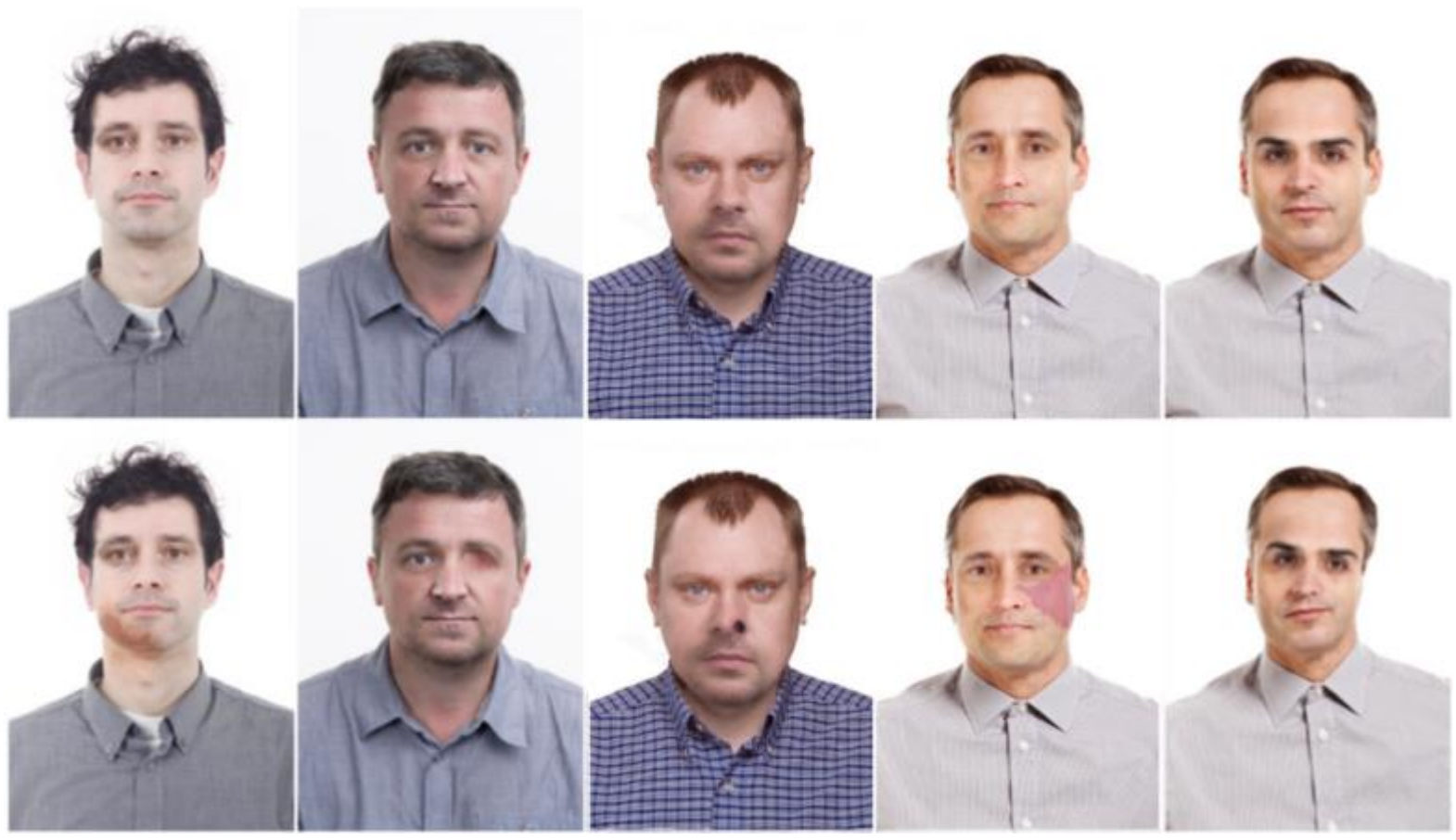

Supplementary Figure 1. Image stimuli used in the IAT. Images on the top row have no facial differences. Images on the bottom row have facial differences. Image Resource: Depositphotos.
